# Co-Created Research Assessing the Exclusionary Impacts of Low Living Standards on Older People

**DOI:** 10.1093/geroni/igaa057.2507

**Published:** 2020-12-16

**Authors:** Charles Waldegrave, Chris Cunningham, Catherine Love, Giang Nguyen

**Affiliations:** 1 Family Centre Social Policy Research Unit, Lower Hutt, Wellington, New Zealand; 2 Research Centre for Maori Health and Development, Massey University, Wellington, Wellington, New Zealand

## Abstract

The aim of this research is to identify the impacts of material resources such as income, assets, housing and living standards on quality of life, health status and social relations. Amartya Sen’s capabilities approach has formed the conceptual basis of the theoretical framework. This paper will report on the results of co-created research with older Māori in New Zealand aged 50 years and older. Objective measures of income, wealth, housing and living standards are compared with a range of scales including overall wellbeing and subjective health status and co-created scales of indigenous loneliness. The results demonstrate significant relationships between material resources and quality of life, health status and other social relations indicators. They quantify the impact material resources have on key indicators of social relations and social exclusion which enables informed and targeted policy interventions for social inclusion.

